# Curcumin Derivatives in Medicinal Chemistry: Potential Applications in Cancer Treatment

**DOI:** 10.3390/molecules29225321

**Published:** 2024-11-12

**Authors:** Joanna Kuzminska, Piotr Szyk, Dariusz T. Mlynarczyk, Pawel Bakun, Izabela Muszalska-Kolos, Katarzyna Dettlaff, Agnieszka Sobczak, Tomasz Goslinski, Anna Jelinska

**Affiliations:** 1 Chair and Department of Pharmaceutical Chemistry, Poznan University of Medical Sciences, Rokietnicka 3, 60-806 Poznan, Poland; imuszals@ump.edu.pl (I.M.-K.); dettlaff@ump.edu.pl (K.D.); asobczak@ump.edu.pl (A.S.); ajelinsk@ump.edu.pl (A.J.); 2 Doctoral School, Poznan University of Medical Sciences, Bukowska 70, 60-812 Poznan, Poland; piotr.szyk@student.ump.edu.pl; 3 Chair and Department of Chemical Technology of Drugs, Poznan University of Medical Sciences, Rokietnicka 3, 60-806 Poznan, Poland; mlynarczykd@ump.edu.pl (D.T.M.); pbakun@ump.edu.pl (P.B.)

**Keywords:** curcumin, cancer, curcumin derivatives

## Abstract

Curcumin, a naturally occurring compound found in the rhizome of *Curcuma* plants, particularly in turmeric (*Curcuma longa* L.), exhibits a broad range of biological activities, including anti-inflammatory, antioxidant, and anticancer properties. Curcumin has demonstrated effectiveness in inhibiting tumor growth, arousing interest for its potential in treating various cancers, such as breast, lung, prostate, and brain cancers. However, the clinical application of curcumin is limited due to its low chemical stability, poor water solubility, and low bioavailability. In response to these challenges, structural modifications of curcumin have been explored to improve its pharmacological properties, including enhanced anticancer selectivity index and bioavailability. This review highlights promising chemical modifications of curcumin that could lead to the development of more effective anticancer therapies. By functionalizing the parent curcumin molecule, researchers aim to create more stable and bioavailable compounds with enhanced therapeutic potential, making curcumin derivatives promising candidates for medical applications.

## 1. Introduction

Curcumin or (1*E*,6*E*)-1,7-bis(4-hydroxy-3-methoxyphenyl)hepta-1,6-diene-3,5-dione, is a compound present in the rhizome of the *Curcuma* genus plants, most notably in turmeric (*Curcuma longa* L.). Historically, curcumin’s vivid color led to its use as a dye, while its flavor made it popular as a kitchen spice. It is also worth remembering that turmeric, containing curcumin, has been used in traditional medicine for many centuries [1]. Curcumin is classified as a diarylheptanoid. Diarylheptanoids are a group of compounds characterized by two aromatic rings connected by a seven-carbon chain. Depending on the type of seven-carbon unit connecting these rings, diarylheptanoids are divided into four subgroups. These include: (i) linear diarylheptanoids, of which curcumin is a representative; (ii) tetrahydropyran diarylheptanoids, which are characterized by a tetrahydropyran ring in the seven-carbon chain, such as centrolobin; (iii) diarylethyl heptanoids, which contain an aryl-arylethyl bond, such as Acerogenin A; and (iv) biphenyl-diarylethyl heptanoids, in which a biphenyl bridge is present (e.g., Acerogenin E). Diarylheptanoids have attracted intense interest from medicinal and synthetic chemists because of their wide-ranging biological activities [2,3,4,5].

**Figure 1:**
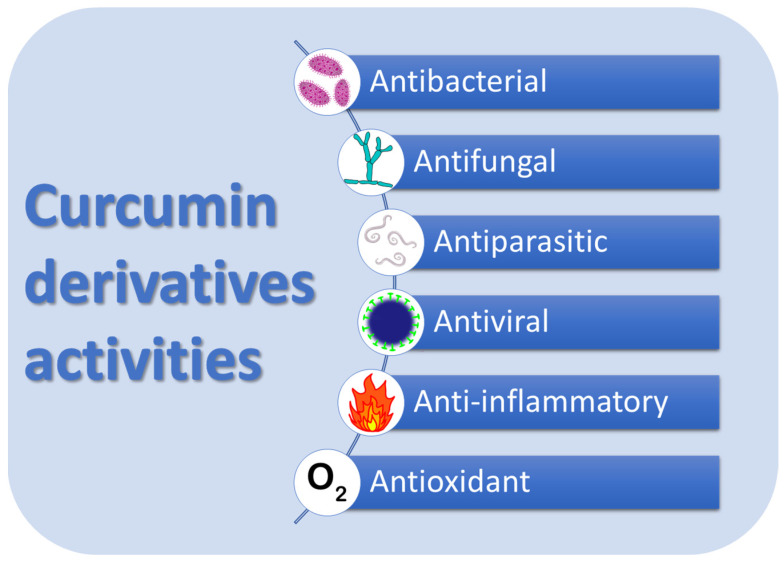
The biological activities of curcumin derivatives.

There is an ongoing interest in the use of curcumin in medicine, especially against cancer and pathogens. Of great importance are its anti-inflammatory and antioxidant properties and potential applications in the treatment of diabetes cardiovascular, and inflammatory bowel diseases (Figure 1) [6]. Biological activities of curcumin are often the results of curcumin promoting or inhibiting certain enzymes and cellular pathways [7,8,9]. This is one of the reasons curcumin exhibits unique anticancer activity, inducing apoptosis and inhibiting tumor growth [10]. Studies have shown curcumin’s effectiveness against many types of cancer, including breast, lung, kidney, uterus, cervical and prostate cancers, squamous cell carcinoma of the head and neck, and brain tumors. Curcumin has also shown potential in suppressing chemoresistance in various cancers. The compound and its derivatives mimic estrogen and compete via aryl hydrocarbon receptors for entry to cells. The benefits of co-delivering curcuminoid derivatives were studied in many breast cancer cell lines (e.g., MDA-MB-468, MDA-MB-231, BT-549, BT-20, and MCF-7). The current understanding of curcumin’s role and that of its derivatives in chemosensitization is based on its multi-sectoral action—reactive oxygen species (ROS) generation, activity modulation of protein kinases, pro-apoptotic regulators, histone deacetylase, telomerase, efflux pumps, and many more [11].

Despite these benefits, the clinical use of curcumin is hindered by its low chemical stability and limited solubility in water, resulting in poor bioavailability after oral ingestion [12]. In addition, rapid elimination from the human body limits the therapeutic use of this compound [13]. The hydrophobic nature of curcumin limits its cellular uptake, as it tends to bind to the fatty acyl chains of membrane lipids rather than efficiently entering the cytoplasm. To overcome these challenges and enhance curcumin’s anticancer activity, its structural modifications are being researched to improve selective toxicity against cancer cells, increase bioavailability, and enhance stability [14,15,16,17,18,19,20,21,22,23,24].

The physicochemical shortcomings of curcumin are associated with the presence of the enol fragment and an active methylene group in its structure. These features are responsible for the low solubility and limited stability of curcumin in the biological media [7,8,9]. Modifications of curcuminoids quite often aim to increase the solubility and improve the compatibility within the enzyme active centers. Two main approaches are used to obtain new curcuminoids: (i) modification of naturally occurring curcuminoids and (ii) synthesis starting from simple precursors. Modifications to the curcumin molecule primarily target the hydroxyl and methoxyl groups, as well as the β-diketone moiety, which undergoes tautomerism to its enol form in an alkaline environment. The hydroxyl groups may be converted to esters, whereas the ß-diketone moiety may undergo condensations or act as ligands in complexes. The active methylene group between two carbonyls is acidic in nature and thus susceptible to electrophilic attack. Synthesis from simple precursors typically involves aryl compounds and functionalized hydrocarbon chains or rings [25,26,27,28,29,30,31]. This review highlights promising modifications of curcumin derivatives with potential anticancer activity. This review attempts to answer two fundamental questions: (i) whether and to what extent curcumin and its derivatives can be used in the therapy of selected cancers, and (ii) in what direction should we proceed in the design of new active curcumin derivatives? For this purpose, the first part covers a review of the literature on the efficacy of curcumin and its derivatives in selected types of cancer, proving the validity of further studies aimed at chemical modification of its molecule. In turn, the second part of the review includes the structure/pharmacological activity relationship of curcumin derivatives. To sum up, the aim of this review is to indicate which cancers have potential for the use of curcumin and its derivatives and in what direction the research on the anticancer activity of newly synthesized derivatives could be conducted.

## 2. Curcuminoid Activity Against Cancers

**Figure 2:**
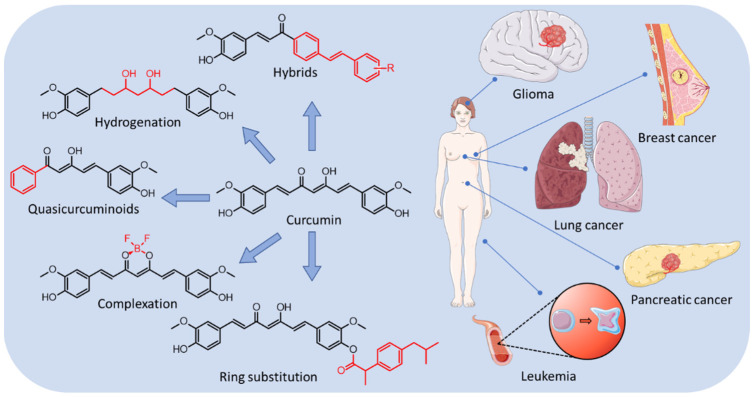
Selected curcumin derivatives and their activity against selected cancer diseases.

In this chapter, selected curcumin derivatives with potential activity against cancer are discussed. In the following subsections referring to several types of cancers, studies on curcuminoid derivatives are presented (Figure 2).

### 2.1. Breast Cancers

In 2022, there were an estimated 20 million new cases worldwide and 9.7 million cancer deaths. For women, breast cancer alone is predicted to contribute to 31% of female cancer cases in 2023 [32]. Breast cancer is clinically categorized into five subtypes depending on the expression of estrogen receptors (ER), progesterone receptors (PR), and the human epidermal growth factor receptor 2 (HER2) oncogene. Tumors that express ER and/or PR are classified as receptor-positive breast cancers, while those lacking ER, PR, and HER2 expression are referred to as triple-negative breast cancers (TNBC). Presently, the primary treatment options for breast cancer include chemotherapy, endocrine therapy, oligo-small molecule inhibitor therapy, and surgical removal of the tumor [33,34]. Curcumin and its derivatives have demonstrated significant efficacy against various cancers. Evidence from in vivo and in vitro studies indicates that curcumin exhibits breast anticancer properties through numerous mechanisms, including induction of cell cycle arrest and apoptosis, modulation of relevant signaling pathways and gene expression, inhibition of tumor cell proliferation, suppression of metastasis, and prevention of angiogenesis. Detailed documentation has shown that the main targets and signaling pathways interacting with curcumin include: nuclear factor kappa-light-chain-enhancer of activated B cells (NF-κB), p53 protein (p53), vascular endothelial growth factor (VEGF), ROS, PI3K/AKT/mTOR pathway, protein kinase B, Wnt/β-catenin, JAK/STAT signaling pathway, ER, HER2, and microRNA. As mentioned before, the clinical use of curcumin and its efficacy is limited due to its unfavorable physicochemical properties despite such promising effects. This chapter reviews recent advances in research on the synthesis of curcumin derivatives, focusing on their action in breast cancer therapy.

**Figure 3:**
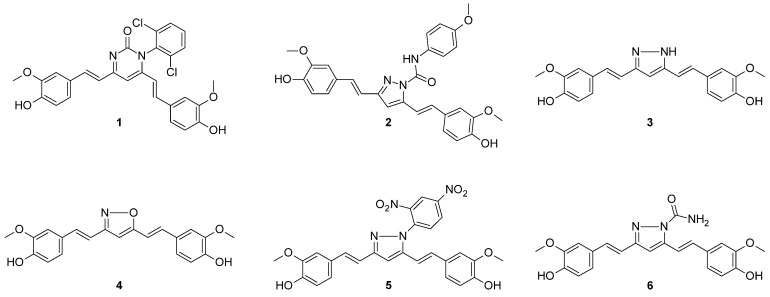
Chemical structures of curcumin derivatives **1**–**6**.

Afzal et al. [29] condensed phenyl urea group with two carbonyl groups of curcumin. The authors obtained three pyrimidinone analogs, among which **1**, visualized in Figure 3, revealed the highest inhibitory activity towards MCF7 (breast cancer cell line). This effect could be assigned to an affinity of **1** to the active site of the epidermal growth factor receptor (EGFR). The phenomenon was further examined by molecular docking studies and led to the observation that compound **1** had the strongest binding affinity to EGFR among the studied compounds, with three different types of interactions. Data on growth inhibitory potential (at the concentration of 10 µM) was collected from nine different types of cancers and 59 cell lines, six of which were breast cancer cell lines. The mean growth inhibitory potential from these six lines was calculated as 75%, which is an improvement compared to curcumin growth inhibitory potential—56% [29]. A pyrazole derivative of curcumin **2** revealed lower mean growth inhibitory percentage points against the same six types of breast cancer cell lines compared to compound **1** [29,35].

Structurally similar compounds were studied by Rodrigues et al. [36], who assessed four five-membered heterocyclic derivatives of curcumin **3**–**6** (see Figure 3) using in silico and in vitro studies on the MCF7 cell line. The retention of characteristic curcumin scaffold, namely the carbonyl chain and the aryl side chain, and a modification of β-diketone moiety played a fundamental role in improving the biological properties. Curcuminoids **3** and **6** were less potent than curcumin, based on IC_50_ values. Although the substituted pyrazole derivative **5** presented a satisfactory IC_50_ value, the compound was less soluble and tended to precipitate. The most potent derivative was **4**, with an IC_50_ value lower than that noted for curcumin but higher than that of **5**. In contrast to **5**, derivative **3** did not reveal any physicochemical shortcomings. Additionally, in silico calculations showed that the absorption from the gastrointestinal tract would be the highest for **3**, and the compound would have a good binding affinity to key proteins that play a role in cancer progression. All things considered, the isoxazole analog was identified as a promising lead structure for further evaluation [36].

**Figure 4:**
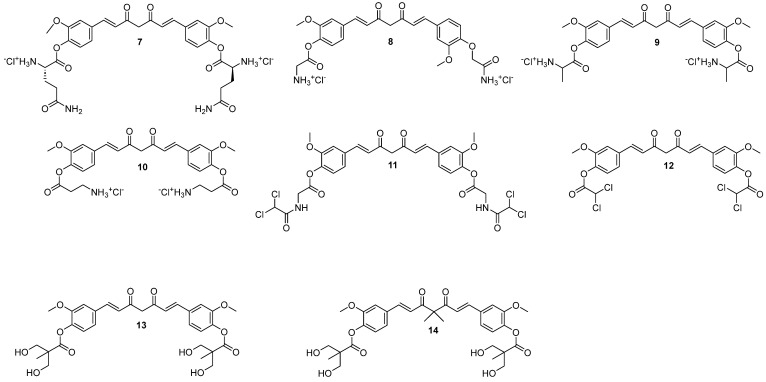
Chemical structures of curcumin derivatives **7**–**14**.

Panda et al. [25] esterified curcumin using amino acids and screened them for anticancer, antimicrobial, anti-inflammatory, and analgesic properties (compounds **7**–**10** in Figure 4). The novel conjugates revealed a promising effect on the MCF7 cells (IC_50_ values between 9.15 and 11.52 µM), more profound than towards lung and prostate cancer cell lines. Interestingly, analogs with protected amines exhibited IC_50_ values exceeding 100 µM [25].

Panda et al. [37] continued their work on esterified curcumin derivatives and, in another article, reported on dichloroacetic derivatives of curcumin (**11** and **12** in Figure 4) conjugated directly via the ester bond or an amino acid linker—glycine, L/β-alanine, L-phenylalanine, or γ-aminobutyric acid. Dichloroacetic acid is a potent anticancer agent that suffers from worrisome toxicity. A total of six novel compounds were used in a clonogenic survival assay, which showed the suppression of the proliferation of T-47D and MDA-MB-231 breast cancer cell lines but not the healthy MCF10A epithelial cells from the human mammary gland. The activity of the compounds was about 8–16 times greater towards cancer cell lines, with EC_50_ values up to a nanomolar level at 424 and 778 nM for **11** and **12**, respectively. Further study of **11** in the mouse mammary tumor model showed significantly reduced tumor volume gain compared to the control group and dichloroacetic acid alone. Moreover, no increased systemic toxicity was observed as the body weight, organ histology, and blood parameters were optimal. Finally, the in silico studies predicted that compound **11** would have a better inhibitory affinity towards DYRK2 (a protein that promotes proliferation) and lower towards hERG (inhibition causes cardiac-related disorders), would be weaker metabolized by CYP2D6 and would not be a P-glycoprotein−P-gp (an efflux pump) substrate [37].

Different kinds of derivatives were obtained by Hsieh et al. [38], of which compounds **13** and **14** were mainly assessed. Both compounds revealed better activity than curcumin. It’s worth noting that hydroxyl groups of curcumin were substituted with dihydroxyacids in a series of reactions to yield **13** (Figure 4). As compared to curcumin, the novel analog showed good stability, higher hydrophilicity, and solubility in water and alcohol, indicating better potential. It was further examined both in vivo and in vitro on mice models and the MDA-MB-231 breast cancer cell line, respectively. The calculated IC_50_ value was 6.1 times lower against MDA-MB-231 than curcumin, and the value was also lower than that obtained for monosubstituted derivative. However, it was slightly higher than that observed for the analog with a longer alkyl chain—bis(hydroxymethyl)butanoic acid. Moreover, swapping the position between ether and ester in the aromatic rings increased the IC_50_ value. Interestingly, elongation of the alkyl group in the swapped ether-ester compound resulted in a decrease of the inhibitory potential, which was a different result than in the non-swapped ether-ester compound. However, all mentioned derivatives in this paragraph were still more potent than the parent curcumin. The effect of tautomerization between keto and enol was also studied. The authors substituted the methylene group in between the carbonyls with two methyl groups—preventing the formation of enol form and concluded that the novel derivative had a lower IC_50_ value. Further evaluation of compound **13** proved a synergistic effect with doxorubicin against the doxorubicin-resistant MDA-MB-231 cancer cell line. Furthermore, administration of **13** to the MDA-MB-231 xenograft nude mice model reduced the tumor size by 60%, whereas in combination with doxorubicin by 80% of that of the control group. Additionally, there was no difference in body weight, behavior, and blood chemistry between treated and untreated mice [38]. The effect could be attributed to G2/M phase arrest, apoptosis, and autophagy of the treated cancer cells, as evaluated in a following study [38,39]. Furthermore, curcumin derivative **13** decreased invasiveness by MDA-MB-231 cells in concentrations below 5 µM by inhibiting the secretion of proteins that cause the degradation of gelatin and collagens, as well as inhibiting the MAPK/ERK/AKT signaling pathway [40]. 

**Figure 5:**
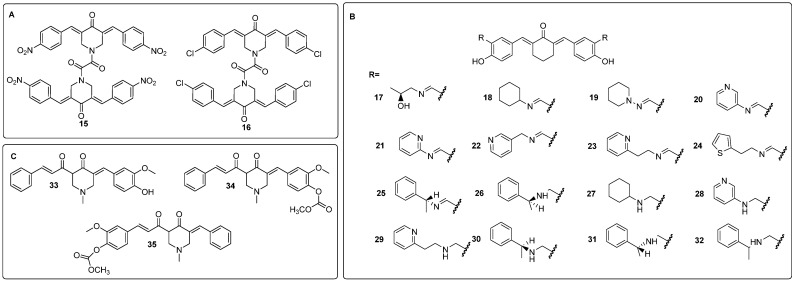
Chemical structures of curcumin derivatives: **15**–**16** (**A**), **17**–**32** (**B**), **33**–**35** (**C**).

Curcumin dimers are known to be more stable and to have better inhibitory potential towards cancer cells. Moreover, curcumin-piperidone derivatives also show better antiproliferative potential on cancer cells. Therefore, a combination of the two modifications was a driving force for studies by Koroth et al. [41] and Nirgude et al. [42]. Two interesting dimers (**15** and **16**) are shown in Figure 5A. Those compounds bear chloro- or nitro- substituents in the aromatic rings, and the hydrocarbon chain between the aromatic rings is shorter. The authors stated that adding an electron-withdrawing substituent (-NO_2_, -Cl) to the parent structure enhanced the antiproliferative potential. Compound **15** was tested in vitro against the MCF7 and metastatic MDA-MB-231 breast cancer cell lines. The effective dose was at the nanomolar lever, namely 54 and 127 nM for MDA-MB-231 and MCF7, respectively. The derivative **15** was 100 times more potent than curcumin and revealed better potential against less differentiated and more metastatic cancer cells. At the same time, it did not show any cytotoxicity against peripheral blood mononuclear cells in concentrations of up to 150 nM. The mechanism of action was via the activation of the intrinsic pathway of apoptosis. In addition, the migration capacity rate of MDA-MB-231 was diminished, and the effect was attributed to the downregulation of the expression of matrix metalloproteinase 1 [41]. Research on compound **15** was continued in subsequent in vivo studies on EAC mice tumor allografts [43]. Compound **15** was effective and demonstrated a synergistic effect with doxorubicin, cisplatin, and olaparib. Simultaneously, derivative **15** was found to be safe as it did not cause any histopathological or body mass changes as compared to the control group. Authors found evidence for the pleiotropic action of compound **15**; there were found 74 and 114 changes in miRNA and mRNA expressions, respectively. The authors also described a unique miRNA-mRNA interaction network, which indicated an impact on the regulation targets of NF-κB [43]. Analog **16** was also evaluated and revealed similar biological properties to **15** with IC_50_ at 31 nM against MDA-MB-231 [42].

Other modifications described in the literature include replacing the β-diketone group with cyclohexanone (which increases the stability and bioavailability) and substituting some hydrogens in the aromatic rings with N-alkyl-methanimines or N-alkyl-methanamines [44]. Both the synthesized imino and amino curcumin substituents (compounds **17-32**, Figure 5B) showed better anticancer potential than curcumin and methotrexate, as the novel analogs had IC_50_ values towards MCF7 in the range of 10–300 μg/mL. The substitution of piperidine (**19**) did not change IC_50_ values as compared to curcumin, but the replacement of the piperidine ring to cyclohexane drastically decreased the IC_50_ value (**18**). The change to the pyridine ring (compounds **20**–**21**) also affected IC_50_, with the values slightly above those for compound **18** but still almost 6 times lower than that for curcumin. In addition, the position of the nitrogen atom in the pyridine ring impacted IC_50_, as compound **20** had lower IC_50_ values than **21**. Compounds with longer linkers between the heterocycle and imine, **22**–**23**, had lower IC_50_ values than those with very rigid structures **20**–**21.** Approximately a two-fold decrease of IC_50_ was observed for S stereoisomers of 1-phenylethan-imine (compound **25**) as compared to the R isomers (compound **26**). Interestingly, the reduction to 1-phenylethan-amine did not change the IC_50_ value a lot, but an opposite relationship in reduced imines could be observed—isomer R (**30**) had lower IC_50_ as compared to S isomer (**31**). Differently, for piperidine and pyridine analogs, the reduced compounds (imine to amine) **27**–**29** showed lower IC_50_. Overall, three compounds, **18**, **27**, and **28** were acknowledged as the most potent in the study, with IC_50_ values lower than that of methotrexate [44].

Kostrzewa et al. [45] developed structurally similar 4-piperidone ring-fused curcumins those exhibited antioxidant or ROS-generating properties, which induced PTP1B enzyme degradation (compounds **33**–**35**, Figure 5C) [45]. The introduction of a nitrogen atom and protection of the hydroxyl group by acetyl groups in curcumin were aimed to increase the cytotoxicity effect and reduce metabolism, respectively. In the nitro blue tetrazolium test, **35** showed the best antioxidant properties, whereas in the in vitro cytotoxicity test on MCF-7 and MDA-MB-231 cell lines, **33** and **34** revealed the best IC_50_ values and were even better than curcumin. Interestingly, structural isomers **34** and **35** showed different properties, namely the isomer with 4-piperidone moiety closer to the non-substituted aromatic ring **35** revealed better antioxidant properties. Compounds **33** and **34** were further evaluated and both showed similar cytotoxicity towards the MCF-7 cell line, but against the MDA-MB-231 cell line, the **34** showed about two times greater inhibitory potential compared to **33**. Thus, the protection of the hydroxyl group played a role in augmenting the anticancer effect on more malignant cell lines. Both **33** and **34** were found to generate ROS in cancer cell lines but not in the HaCaT cells. Compound **33** was evaluated as a photosensitizer in MDA-MB-231, and the results indicated that the compound showed higher cytotoxicity after irradiation with green light compared to curcumin. The in silico studies revealed that the inhibition of PTP1B could be caused by allosteric regulation by **34** [45].

An analysis of the reviewed studies allows us to identify how particular changes in structures of discussed compounds impact their biological activity, bioavailability, and stability.

– Hydroxyl and methoxyl groups modifications:
  - The esterification at the 3′-hydroxyl group is favored over esterification at the 4′-hydroxyl group in terms of biological activity [38].
  - Substituents with longer alkyl chains are more effective at the 4′-hydroxyl position compared to the 3′-hydroxyl position [38].
  - Asymmetric ester derivatives of curcumin should be prioritized for consideration, as some demonstrate higher potency compared to symmetric modifications of the hydroxyl groups [38].
  - Amino acid derivatives protected with Boc, Fmoc, and Cbz groups are generally ineffective unless these protective groups are replaced with dichloroacetic acid [25,37].
  - The introduction of amines or imines has been shown to enhance activity, particularly those featuring pyridine or piperidine rings [44].
– ß-Diketo moiety adjustments:
  - Conversion to a pyrimidone ring enhances EGFR targeting properties [29].
  - Isoxazole ring exhibits greater potency compared to pyrazoles [36].
  - Dimers of piperidinone-modified curcumin demonstrate increased efficacy [41,42,43].
  - Conversion to cyclohexanone improves bioavailability and stability [44].
– Ring modification:
  - The electron-withdrawing groups (EWGs), such as nitro (NO_2_) and chlorine (Cl), correlate with enhanced anticancer activity [41,42].

### 2.2. Glioma

Glioblastoma multiforme has a low survival rate due to frequent recurrence and resistance to current treatments, which is largely due to the molecular heterogeneity of gliomas and the tumor microenvironment. Communication between glioma cells, healthy cells, and the immune system promotes cancer progression and resistance to treatment, particularly through the development of glioma stem cells. In addition, factors released by the tumor and environmental influences, such as hypoxia, help cancer cells evade detection by the immune system and promote disease progression. Curcumin inhibits the growth of malignant gliomas by affecting various cellular processes, including proliferation, apoptosis (through downregulation of bcl-2, bcl-xL, and activation of caspases), autophagy, angiogenesis, immunomodulation, as well as invasion and metastasis. In particular, curcumin has been found to selectively target and kill cancer cells while non-cancerous nerve cells such as astrocytes and neurons. In addition, it can trigger autophagy, which is regulated by simultaneous inhibition of the Akt/mTOR/p70S6K pathway and activation of the ERK1/2 pathway. Overall, these findings underscore the anticancer potential of curcumin, as well as its analogs, which may exhibit better activity and bioavailability than curcumin alone [46,47,48].

**Figure 6:**
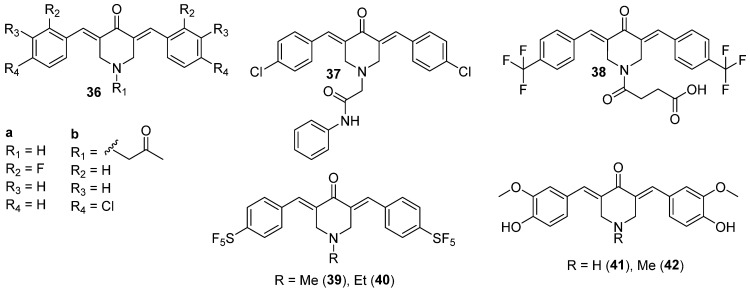
Chemical structures of curcumin derivatives **36**–**42**.

The previously described compound **1** (Figure 3) also had potent activities against CNS cancer cell lines, stronger than curcumin. Specifically, the growth inhibition was above 80% for SF-268, SF-539, and U251 and below 50% for SF-295 [29]. Apart from using a pyrimidin-2-one ring in exchange for the diketo group of curcumin, a series of piperidin-4-one analogs was also explored in this matter. Huber et al. [49] synthesized novel C5-curcuminoids to be tested on glioma cell lines and subjected to blood-brain barrier permeation studies [49]. Three of them (compounds **36**–**38**), with promising properties, are shown in Figure 6. The C5-curcuminoids exhibited better stability, and the introduction of a ring in the alkyl part of the compound made the structure more rigid. Moreover, the aryl rings were p-substituted by halogen/alkylhalogen or by hydrogen, as that kind of modification could improve cytotoxicity. For **37**, the authors exploited a known motif from lidocaine—a weakly basic nitrogen atom that enhances blood-brain barrier permeation and for **38**, a carboxylic group was introduced to exploit the function of monocarboxylic acid transporters. In the in vitro study, **36** and **38** were the most potent against astrocytoma and neuroblastoma, respectively (IC_50_ below 1 nM). Interestingly, the cytotoxic effect did not exponentially rise with increasing a dose of these compounds, which might indicate some saturation of targets. The major concern refers to its toxicity towards healthy cells, which cannot be fully avoided, but curcumin derivative **38** revealed the best selectivity against neuroblastoma compared to kidney cells. The trifluoromethyl substitution present in the chemical structure of **38** was labeled as the most promising compared to methoxy or halogenic substitution. Overall, the most potent compounds were those with monocarboxylic substituted nitrogen in the piperidin-4-one ring. No clear correlation could be found between lipophilicity/solubility and cytotoxicity, but the study provided evidence that some compounds undergo the thia-Michael reaction effect that could increase solubility [49]. In terms of blood-brain barrier (BBB) permeability of **36**–**38**, It was found that **37**, the most insoluble in water of the three, showed the best permeability, which is consistent with the fact that lipophilic substances cross the BBB [50].

Structurally similar curcumin derivatives, including pentafluoro-substituted compounds, were used to target the stem-like phenotype of glioma cells, which is responsible for cancer recurrence [27]. The introduction of the electronegative pentafluorothio group revealed a larger impact on bioactivity than on the fluoro moiety in the rings, as **39** and **40** (Figure 6), especially in terms of antiproliferative and antiangiogenic activities. The cytotoxic effect of **39** was up to ten-fold greater than fluorinated analog **36a**. In all tested cells, including U251 and Mz54 glioblastoma cell lines, methylated analog **39** was also more potent than the ethylated one **40**. Both **39** and **40** decreased the sphere-forming capacities of the glioma—stem-like cell sphere cultures, along with IC_50_ at nanomolar concentrations. In addition, the novel compounds were more selective toward cancer cells than to endothelial hybrid cell lines (EA.hy926) [27]. In turn, the activity of curcumin-piperidin-4-one derivatives **41** and **42** (Figure 6), differing from curcumin only in the diketo fragment, was evaluated on the LN-18 human glioblastoma cell line [51]. Both **41** and **42** showed greater antiproliferative potential in the cell culture than curcumin. The IC_50_ values towards healthy cells were around 2.3 times higher than for LN-18, indicating the selectivity of the compounds toward cancer cells. Besides the cytotoxic effect, the authors found that **41** and **42** had an anti-migratory effect, and 41 additionally presented an anti-invasion effect. The tested analogs caused cell cycle arrest of LN-18 in the SD phase, while for curcumin, the effect was noted in the G2/M phase [51].

**Figure 7:**
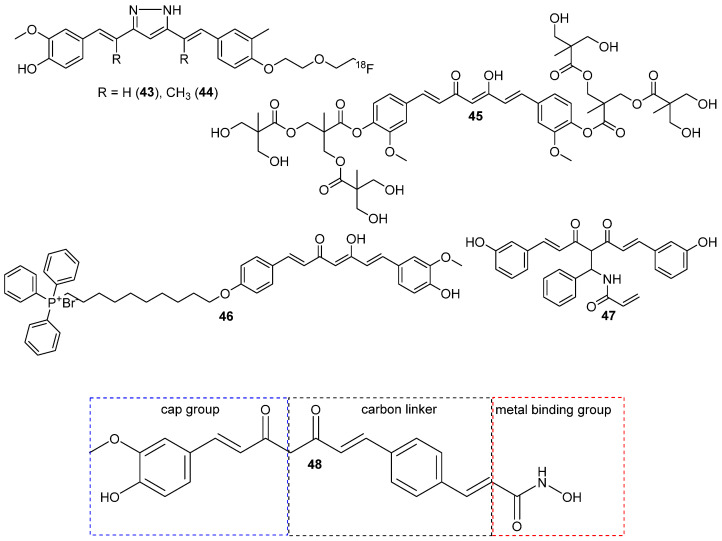
Chemical structures of curcumin derivatives **43**–**48**.

A slightly different approach (which was successful for breast cancer) for curcumin derivatization was reported by Shin et al. [52]. The two novel compounds were equipped with a conjugated ring in the alkyl region between the rings and side chain with ^18^F. Positron emission tomography imaging of C6 glioma xenografted mice indicated the highest uptake in tumor tissue for **44**, but both tumor-to-blood or to-muscle ratios of **43** and **44** were nearly the same (Figure 7) [52].

Further modifications of curcumin, involving linking its hydroxyl groups in the para position with a second-generation polyester dendron, were presented by Landeros et al. [28]. The *para*-hydroxyl groups were coupled with first-generation polyester dendron leading to compound **45** (Figure 7). This modification did not cause a loss of antioxidant properties, whereas an improvement in solubility was significant. Compound **45** was acting in an antiproliferative manner towards C6 glioblastoma cells at lower concentrations than curcumin and simultaneously was less cytotoxic towards healthy NHDF cell lines. Some differences in its mode of action were also observed, as cell death was caused rather by necrosis or autophagy. As the uptake was compared to curcumin, **45** was internalized in less amount in the first 6 h, but after 24 h, this reversed, and more compound **45** was internalized [28].

Shi et al. [53] synthesized curcumin derivative conjugated with triphenylphosphonium cation through the alkyl chain as a linker—**46** (Figure 7). This approach allowed for its greater accumulation in mitochondria and decrease of thioredoxin reductase (Trx) enzyme activity, especially the isoform Trx2. The inhibition of Trx2 was a contributing factor disturbing redox homeostasis, which led to ROS generation and further activation of caspases and intrinsic apoptosis. In addition, disturbed mitochondrial respiration by reduction by half in basal respiration, and a reduction of ATP production in the presence of **46** was observed. These effects were not noted or were only marginal for the parent curcumin. Among the six types of cancer cell lines tested, the glioma cell line was the most sensitive. The continuation of in vitro research on various glioma cell lines indicated that temozolomide-resistant glioma cell lines are susceptible to **46**. In the final part of the research, an in vivo antitumor activity evaluation was performed on a mouse model, which confirmed a better therapeutic outcome for **46** compared to curcumin [53]. This is consistent with the effects observed for similar derivatives containing triphenylphosphonium cation as the targeting moiety bound to the curcumin scaffold [54].

Another curcumin analog researched in vitro and in vivo on glioma models was **47** (also known as C-150) [30]. In the chemical structure of **47**, one of the hydrogens from between the carbonyl groups was substituted with N-(1-phenylethyl)acrylamide (Figure 7). This led to reduced transcriptional activation of NF-κB and inhibited PKC-alpha kinase, both proteins implicated in gliomas, with seemingly no effect on mTOR or AKT1. The compound **47** was more cytotoxic in at least eight glioma cell lines and had 26 times lower inhibition values of NF-κB compared to curcumin. Moreover, in vivo studies revealed that **47** inhibited the formation of tumors in a special mutant strain of *Drosophila* and prolonged the median survival time of a rat model with intracerebrally implanted glioblastoma cells [30].

Based on the structure of the currently approved histone deacetylase (HADAC) and molecular modeling, Wang et al. [55] modified one of the aromatic rings to increase curcumin inhibitory potential toward HADAC. The methoxy group was removed, and the hydroxyl group was changed to N-hydroxyacrylamide [55]. Following molecular modeling, the novel compound consists of three major regions (Figure 7): (i) the cap group is exposed to the solvent space and interacts with the rim of the catalytic tunnel; (ii) the metal binding group occupies the catalytic site, and (iii) the carbon linker connects the two parts and interacts with phenylalanine through π–π stacking. Compound **48** revealed greater inhibition potential in vitro against some isoforms of HADAC, but it was a bit lower compared to vorinostat, an FDA-accepted HADAC inhibitor. The IC_50_ value was lower for **48** compared to curcumin and higher compared to vorinostat. The derivative was more resistant to metabolism, as the stability was five times higher in human liver microsomes than in curcumin. The in vivo study on mice showed the T1/2 of 3.2 h after oral dosing, with a bioavailability of 40.2%. Blood-brain barrier permeability was low but acceptable, with brain-to-plasma ratios of 0.08–0.23. It was also established that **48** caused cell apoptosis and cell cycle arrest in phase G_2_/M. In vivo comparison to vorinostat in mice with subcutaneously inoculated U87 cell line cells revealed no observable toxicity for either of them, yet **48** inhibited the tumor growth twice as much [55].

The reviewed literature provides insights into how certain structural modifications impact the physicochemical properties, in vivo behavior, and anticancer efficacy of the above-discussed curcumin analogs.

- Hydroxyl and methoxyl groups modifications:
  - Polyester dendrimeric substitutions enhance both solubility and activity [28].
  - Triphenylphosphonium cation increases the mitochondrial accumulation of curcumin derivatives [53].
- ß-Diketo moiety adjustments:
  - Pyrimidinone modifications result in improved anticancer activity [29].
  - The addition of N-phenyl amides and carboxylic acids enhances both blood-brain barrier permeability [49] and antiglioma activity [30,49].
  - N-Methyl substituted piperidinones exhibit greater efficacy than N-ethyl derivatives [27], with non-substituted variants being more effective than substituted ones [51].
- Ring modifications:
  - Trifluoromethoxy substitution at the 4′ position significantly increases cytotoxicity compared to hydrogen, chlorine, and fluorine substitutions [49].
  - Pentafluorothio substituents at the 4′ position demonstrate greater efficacy than 2′-fluorine substituents [27].
  - Asymmetric curcumin derivatives, featuring one unchanged ring and one phenyl ring with p-substituted N-hydroxyacrylamide, effectively inhibit histone deacetylase [55].

### 2.3. Pancreatic Cancer

Pancreatic cancer ranks as one of the leading causes of cancer-related deaths globally, with its incidence more than doubling over the past 25 years. The most affected regions include North America, Europe, and Australia. While this rise is largely driven by an aging global population, several modifiable risk factors—such as smoking, obesity, diabetes, and alcohol use—significantly contribute to the disease. The increasing prevalence of these risk factors in many parts of the world is causing a rise in the age-adjusted incidence rates of pancreatic cancer. However, the extent to which these risk factors contribute varies across different regions due to differences in their prevalence and the effectiveness of prevention strategies. Pancreatic cancer, often referred to as the “silent killer,” poses a significant challenge in cancer treatment. The PI3Kα signaling pathway’s dysregulation in pancreatic cancer has become a focal point for therapeutic strategies. As a result, curcumin derivatives have gained attention as potential PI3Kα inhibitors, offering a promising new approach to developing effective treatments for this aggressive disease [56,57].

**Figure 8:**
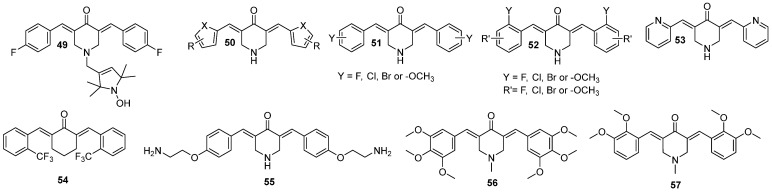
Chemical structures of curcumin derivatives **49**–**57**.

In order to better understand the mechanism of action of known curcumin derivative **49** (also known as HO-3867, Figure 8), Hu et al. [58] studied its effect on PANC-1 and BXPC-3, pancreatic cancer cell lines. The antiproliferative activity towards these cell lines was confirmed, and the authors noted the change in the levels of cell apoptosis-related proteins—a decrease in Bcl-2 and procaspase 3 and an increase in the cleaved PARP protein. At the same time, no changes in Bax expression were noticed. The activity of **49** was correlated with an increased level of ROS generation. Moreover, the augmented level of endoplasmic reticulum stress-related proteins was found. The generation of ROS played a major role in cell apoptosis, as the addition of a ROS scavenger abrogated the decrease in Bcl-2 levels and cell apoptosis. The remaining apoptotic effect was correlated with inhibition of P-STAT3, a protein implicated in resistance to inducing cancer cell apoptosis. In this case, the inhibition did not decrease with the addition of ROS scavenger. Taken together, the authors found two independent **49**-mediated apoptosis pathways [58].

The mentioned pentafluorothio analog **41**, synthesized by Linder et al., revealed strong inhibitory potential against Panc-1 with IC_50_ in nanomolar concentrations, but the inhibitory potential against kappa B kinase β (IKKβ) is unknown [27]. Xie et al., based on two known derivatives in literature, namely EF24 (later discussed in subchapter 3.2.) and EF31 (compound **53**, Figure 8), synthesized a series of analogs (**50**–**52**) (Figure 8). The rationale for the study was to obtain compound with strong inhibitory potential against IKKβ, which is a protein involved in pancreatic cancer development and progression [59]. In the study, the piperidin-4-one ring was recognized as playing a pivotal role in the inhibition. Therefore, a series of derivatives substituted with halogen or methoxy group was obtained. It is worth noting that some of them, fluorinated or brominated, showed good inhibitory activity [59]. Another series, represented by **54**, revealed weaker inhibitory potential than the previous one, with the strongest activity noted for derivatives with fluorine and bromine in the ortho positions [60]. Halogen-substituents introduced a stronger inhibitory effect compared to methoxy. The final series was equipped with bulky substituents on the aryl rings. The most potent derivative **55**, substituted with phenoxyethanamine, based on the collected data, could likely be a direct inhibitor of IKKβ. Molecular modeling gave some clues about the nature of the binding. The rings were squeezed between nine hydrophobic amino acids and the dimethylaminoethoxy groups were oriented toward solvent areas. Both the protein and the compound changed conformation to adjust to each other. In the in vitro studies on three pancreatic cancer cells—Panc-1, MiaPaCa-2, and BxPC-3 the inhibitory effect towards IKKβ was reconfirmed, and the antiproliferative potential was measured. As the outcome of the study, **56** was denoted as a potent compound against pancreatic cancer, with lower IC_50_ values for almost all the cell lines compared to the parental compounds [59].

Chen et al. investigated **54** (Figure 8), also known as C66 curcuminoid, for its antiproliferative potential towards pancreatic cancer cells. In the study, the authors confirmed, by knocking down corresponding genes, that c-Jun N-terminal kinase plays an important role in the proliferation of pancreatic cancer cell lines. Moreover, the expression of the kinase was related to enhanced activity of pro-inflammatory factors. The inhibition of the kinase phosphorylation by **54** was confirmed in the study and further in vitro antiproliferative, anti-migration, and antimetastatic properties of the compound were evaluated. The IC_50_ levels of its activity on Panc-1 and SW1990 were 113.4 and 91.83 μM, respectively [60].

Pignanelli et al. [61] synthesized piperidin-4-one derivatives of curcumin and screened cancer and healthy cells. Two compounds, **55** and **57** (Figure 8), demonstrated some promising properties and were further evaluated. The compounds contributed to intracellular ROS generation and induction of apoptosis. One aspect of the research involved the combinatory action of **56** with piperlongumine on the BxPC-3 pancreatic cancer cell line. The results indicated that **56** acted synergistically with piperlongumine in terms of ROS generation and induction of apoptosis. Importantly, this effect was insignificant in healthy cells [61].

**Figure 9:**
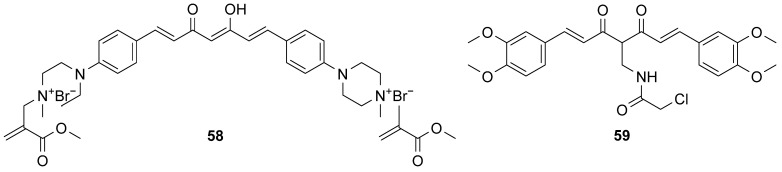
Chemical structures of curcumin derivatives **58**–**59**.

Novel curcumin-derivatized quaternary ammonium salts were considered as another option in the research against pancreatic cancer [31]. One of them, **58** (Figure 9), presented the lowest IC_50_ value on MIAPaCa-2, a pancreatic cancer cell line, compared to that on breast cell lines. The idea that underpinned the derivatization was to join Baylis–Hillman reaction bromide allylic products and quaternary ammonium curcuminoids to utilize the advantages of these two compounds. On the other hand, curcumin showed some anticancer activity, but the low stability and solubility in water did not allow further application. On the other hand, quaternary ammonium curcuminoids had good water solubility, but their cytotoxicity was low, exceeding 100 µM. The change of methyl group from the ester moiety of **58** to longer alkyl groups decreased cytotoxicity. The simplification of the N-substituent to allyl or benzyl strongly decreased cytotoxicity. The authors also attempted to replace the quaternary ammonium curcuminoid skeleton with *N*-methylmorpholine, which again was proven unsuccessful. Compound **58** was further evaluated in vivo on mice, where its safety was confirmed, and MIAPaCa-2 xenograft tumors (growth decreased by 42% or by 57% when used in combination with gemcitabine) [31].

Szebeni et al. [62] synthesized 33 novel curcuminoids and screened them in terms of antiproliferative activity on liver, lung, and pancreas cancer cell lines. Derivative **59** (Figure 9) was chosen as the most promising compound. Interestingly, the novel compounds revealed the strongest effect on the PANC-1 cell line. Derivatives that were equipped with carboxylic, dihydroxyphenyl, or *para*-hydroxyl substituents in the carboxy side of the amide moiety were listed as non-active, while analogical structures with chloroacetamidomethyl moiety and with hydroxyl or methoxyl groups in the rings also had good activity, all of which was in accordance with SAR studies performed by the authors. Curcumin derivative **59** was found to accumulate in the endoplasmic reticulum and induced endoplasmic reticulum stress, which was studied by an up-regulation of genes helping to counteract the stress effect. In comparison to curcumin, the up-regulation was stronger. Furthermore, mitochondrial membrane depolarization and induction of apoptosis were noted.

An interesting effect of curcumin and its cyclohexanone analogs was discovered accidentally by Revalde et al. [63]. During optimization of the liposomal formulation of gemcitabine with curcumin, the authors found that the interaction of these two compounds was antagonistic. Further evaluation on MIA PaCa-2 and PANC-1 cells showed that the curcuminoids inhibited equilibrative nucleoside transporter 1 at concentrations between 2–20 μM and blocked the accumulation of gemcitabine and uridine. The authors concluded that curcumin is unlikely to inhibit gemcitabine uptake in tumors but might have an impact on gastrointestinal absorption. Interestingly, the EF24 (compound **93**, Figure 15) analog did not show this effect. In addition, only co-treatment caused this effect, and the sequential administration did not affect the activity of the transporter [63].

Based on the structure-activity relationship analysis of curcumin derivatives presented in the works cited above, the following conclusions can be drawn.

– ß-Diketo moiety alterations:
  - Pyrimidinone modifications not only enhance anticancer activity [58,61] but also contribute to reactive oxygen species generation [61].
  - The piperidin-4-one ring plays a crucial role in inhibiting IKKβ kinase [59].
  - Derivatives in which acidic hydrogens are replaced with methylamine and further substituted with groups such as carboxylic acid, dihydroxyphenyl, or parahydroxylphenyl exhibit decreased cytotoxicity, while amide formation from 2-chloroacetate increases cytotoxicity [62].
– Ring modifications:
  - Five-membered heterocycles containing oxygen, nitrogen, or sulfur reduce IKKβ kinase inhibition, even when the rings are methyl-substituted [59].
  - Halogenated rings, particularly those with fluorine or bromine, enhance IKKβ kinase inhibition [59].
  - The pentafluorothio- substitution generates strong inhibitory potential against Panc-1 cells, though its impact on IKKβ kinase remains unknown [27].
  - Alkylamine substituents are recommended for consideration when designing IKKβ kinase inhibitors [59].
  - Quaternary ammonium curcuminoids, despite their good solubility, exhibit low cytotoxicity [31].

### 2.4. Other Cancers

This subchapter briefly summarizes recent studies on the potential efficacy and mechanisms of action of curcumin and its analogs against five common cancers, such as colorectal, kidney, lung, and bladder cancer, as well as leukemia.

Colorectal cancer, the third most common cancer worldwide, begins in the large intestine and can spread to the lower gastrointestinal tract. Its development is influenced by genetic mutations, lifestyle factors, and poor diet. Colorectal cancer carcinogenesis involves three key mechanisms: chromosomal instability, CpG island methylator phenotype, and microsatellite instability. Among environmental factors, dietary habits leading to obesity and high energy intake are important risk factors for colorectal cancer. The treatment of colorectal cancer (CRC) is influenced by several factors, such as the stage of the disease, tumor location, and the patient’s overall health. Standard treatments include surgery, chemotherapy, radiation therapy, and immunotherapy, with surgical removal of the tumor (via open or laparoscopic methods) being the primary treatment approach. These therapies are generally more effective when the cancer is diagnosed early, offering around a 90% survival rate. However, late-stage detection leads to a poorer prognosis, with survival rates dropping to approximately 15% in stage IV, highlighting the need for better detection methods and more effective treatments. Currently, chemotherapy remains the cornerstone of CRC treatment, particularly in metastatic cases. Treatment often involves combinations of fluoropyrimidines, such as 5-fluorouracil (5-FU) or capecitabine (CAPE), alongside irinotecan (IRI) or oxaliplatin (OXA), sometimes supplemented with cetuximab for patients with wild-type (wt) RAS or bevacizumab (BEVA). However, overall survival (OS) rates beyond five years are still under 15%. Curcumin, a natural compound, has been extensively studied as a potential therapeutic agent for CRC due to its demonstrated anticancer effects in both in vitro and in vivo models, as well as its low toxicity profile. Curcumin promotes cancer cell death by increasing ROS generation, which activates both intrinsic and extrinsic apoptotic pathways. Research suggests that curcumin enhances the expression of pro-apoptotic proteins like Bax and Bak while inhibiting anti-apoptotic proteins regulated by NF-κB, such as Bcl-2, Bcl-xL, XIAP, and Survivin. This leads to cytochrome c release and the induction of apoptosis. Curcumin’s anti-inflammatory and anticancer properties are largely attributed to its ability to inhibit the NF-κB pathway, which is frequently overactive in CRC and contributes to drug resistance. Beyond its role in apoptosis, curcumin also hinders CRC cell proliferation by influencing cell cycle regulation, inducing arrest at either the G0/G1 or G2/M phases. This is achieved by upregulating cyclin-dependent kinase (CDK) inhibitors like p16, p21, and p27, while inhibiting CDK2, CDK4, cyclin B, E, and D1. Additionally, curcumin reduces proliferation by suppressing cyclooxygenase-2 (COX-2) expression through the NF-κB pathway and modulating AMP-activated protein kinase (AMPK)-AKT signaling. It also affects the Wnt/β-catenin and Notch pathways, which are frequently altered in CRC cases. Despite its promising effects, curcumin’s low bioavailability and unknown interactions with other anticancer drugs limit its therapeutic potential, prompting the search for curcumin analogs with improved physicochemical properties for CRC treatment [64,65,66,67,68].

**Figure 10:**
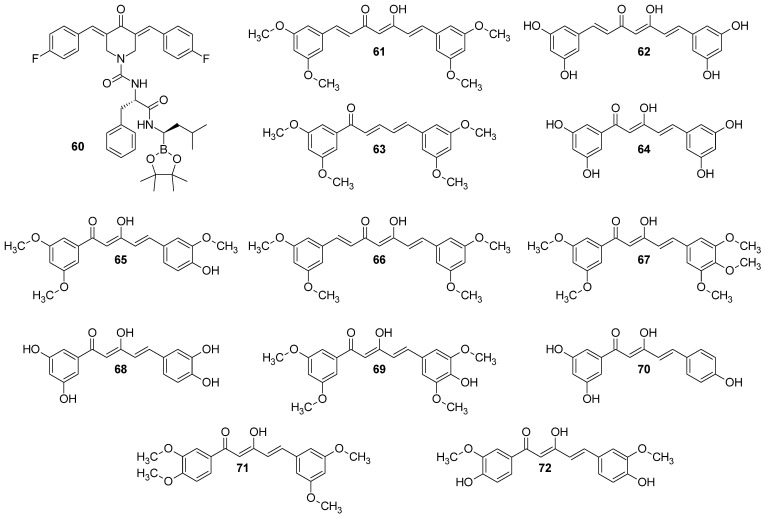
Chemical structures of curcumin derivatives **60**–**72**.

Zhang et al. [69] synthesized two series of curcumin derivatives in order to develop a potent chymotrypsin-like subunit of 20S proteasome, which is one of the regulators of basic cellular processes and pathways, including cell cycle, apoptosis, or DNA repair. All the obtained derivatives showed inhibition of the HCT116 (human colorectal carcinoma) cell line. Among them, **60** (Figure 10) showed excellent inhibition of the chymotrypsin-like activity of the 20S proteasome (IC_50_ = 0.835 µM), with simultaneously only a negligible effect on the other subunits—trypsin-like and peptidylglutamyl-peptide hydrolyzing [69].

Kidney cancer, also known as renal cell carcinoma (RCC), constitutes a group of malignant tumors originating from the epithelium of the renal tubules. RCC is the most common malignant tumor of the kidney, accounting for about 90% of cases. Histologically, about 80% of these tumors are clear cell carcinomas. Kidney cancer ranks as the 15th most frequently diagnosed cancer globally, with a notably higher occurrence in developed countries. The causes of RCC are not precisely known, but risk factors include genetic predisposition (e.g., von Hippel-Lindau syndrome), smoking, hypertension, obesity (especially in women), exposure to certain chemicals, and end-stage renal failure requiring dialysis. RCC accounts for 2–3% of all malignancies, occurring most often in people aged 60–70. Men have the disease 1.5 times more often than women. About 270,000 new cases of RCC are diagnosed annually worldwide, 116,000 of which end in death. The highest incidence rates are seen in Europe, North America, and Australia, and the lowest in Asia and Africa. In Europe, the incidence is 14.5 per 100,000 men and 6.9 per 100,000 women [70]. Curcumin and its derivatives were found to be involved in several pathways associated with the induction of renal cancer cell death and their activity. One of them concerns the modulation of ROS levels. Curcumin and its analog EF24 (later discussed in subchapter 3.2.) were able to increase the activity of peroxidase and, in this way, decrease intracellular ROS levels. Further, this effect led to a decrease in renal cancer cell migration by inhibition of collagenases/gelatinases activity [71]. Chong et al. [72] also reported on the suppression of one of the collagenases by curcumin. This effect of curcumin was combined with the inhibition of transcription factor—E Twenty-Six-1 and impacted by downregulation of the expression of the vascular endothelial cadherin. Both effects resulted in the impairment of the tumor’s ability to ensure blood support by vasculogenic mimicry. Other studies reported the resensitization of renal cell carcinoma when curcumin was used in combination with well-established anticancer drugs. Xu et al. [73] tested combinatory therapy of curcumin and sunitinib and noticed a reversal of sunitinib resistance. The effect was associated with curcumin’s ability to upregulate the ADAMTS18 gene and associated with the induction of ferroptosis [73]. Another study by Xu et al. [74] also indicates that curcumin upregulates the ADAMTS18 gene. However, an additional effect of curcumin was noted, namely upregulation of miR-148 expression. Both effects were associated with the suppression of autophagy and a positive feedback loop between the two genes was proposed [74]. Further study by Xu et al. [75] revealed that the ADAMTS18 gene was upregulated through the downregulation of its methylation by AKT and NF-κB signaling pathway. Obaidi et al. [76] found that curcumin reverses kidney cancer cells’ resistance toward TRAIL (tumor necrosis factor (TNF)-related apoptosis-inducing ligand). This was attributed to curcumin’s deregulation of miRNA expression associated with apoptosis regulation, especially let-7C. This contributed to the downregulation of the expression of cell cycle protein, and furthermore, two key glycolysis-regulating proteins were also found to a lesser extent [76]. Chang et al. [77] also found that curcumin had an impact on miRNA expression, which caused cancer cell death. It was also found that treatment of SK-NEP-1 cells with curcumin led to increased expression of miR-192-5p, whose native role is to downregulate the expression of PI3K and AKT proteins.

Leukemia ranks as the 13th most common cancer and the 10th leading cause of cancer-related deaths globally, with over 487,000 new cases and 305,000 deaths estimated in 2022. The highest incidence rates are observed in Australia/New Zealand (with Australia having the highest rates among men worldwide), Northern America, and various regions of Europe (with Belgium leading among women). The incidence of leukemia is two to three times higher in developed countries compared to developing ones for both men and women, although mortality rates are similar, particularly among women. Leukemia encompasses a diverse group of hematopoietic cancers with distinct biological subtypes, generally classified into four major categories, each with varied causes, including genetic factors, infections, and enhanced diagnostic capabilities. Acute lymphoblastic leukemia (ALL) is more prevalent in children and exhibits a bimodal pattern, with higher incidence rates in Latin American and Asian countries. Acute myeloid leukemia (AML) is more common in adults but also affects children, with higher incidence rates in countries with a higher Human Development Index (HDI). Chronic lymphoid leukemia (CLL) is more frequent among the elderly and males, with elevated rates in North America, Oceania, and parts of Europe. In contrast, chronic myeloid leukemia (CML) is more commonly observed in adult males in higher HDI countries [32].

To assess the efficacy of curcumin in leukemia treatment, a series of studies, both in vitro and in vivo, have been conducted. The literature contains numerous reports on the potential anticancer activity of curcumin as well as its derivatives against various types of leukemia (such as AML, CML, CLL, and ALL). The activities of curcumin and its derivatives were related, among others, to inducing apoptosis, inhibiting proliferation, ROS production, or stimulating autophagy. The mechanism of anticancer action is multidirectional and includes multiple cellular and molecular targets and pathways. The main molecular targets of curcumin in leukemia cancer cells include receptors (e.g., DR-4, DR-5), transcription factors (e.g., Notch-1, NF-κB, STAT3 and 5), kinases (e.g., ERK, JAK), growth factors (e.g., VEGF), inflammatory cytokines (e.g., IL), and others (e.g., HSP-90, Bcl) [78,79,80].

Due to the low potency of curcumin, higher doses are required to achieve a therapeutic response in leukemia, which increases the risk of adverse effects and reduces patient compliance. To address these limitations, various derivatives have been synthesized, and combination therapies have been explored. An example would be the combination of curcumin with plant compounds such as quercetin [81] or cannabidiol with quercetin [82] or with chemotherapeutics like thalidomide [83] or imatinib [84]. In all cases, the co-administration of two or more compounds was shown to enhance the effectiveness of therapy compared to monotherapy. Except for the last one, all mentioned research involved in vitro or in vivo tests on mice models. The final study was a randomized controlled trial conducted on fifty CML patients, who were treated for 6 weeks with imatinib alone (800 mg per day) or imatinib and curcumin (800 mg per day and 15 g per day, respectively). A significant decrease in plasma NO levels and better hematological response and tolerance after a combination of imatinib and curcumin therapy, as compared to imatinib therapy alone, was demonstrated. Based on the studies, the authors concluded that curcumin can be used as an adjuvant to imatinib therapy due to its prominent anti-neoplastic activity [84].

In the context of combined therapies, Zhang et al. [16] presented intriguing findings. They demonstrated an interaction between curcumin and interferon signaling pathways, which could potentially provide the theoretical basis for a curcumin-interferon combination in anticancer therapies. Curcumin has been shown to induce the expression of interferon regulatory genes, particularly IFIT2, in U937 leukemia cells. The regulation of IFIT2 by exogenous expression or IFNγ treatment in K562 cells increased cell apoptosis and enhanced the anticancer effects of curcumin. Conversely, shRNA-mediated IFIT2 knockout inhibited curcumin-induced apoptosis in U937 cells. These findings open up new avenues for research and potential future combined treatments. 

**Figure 11:**
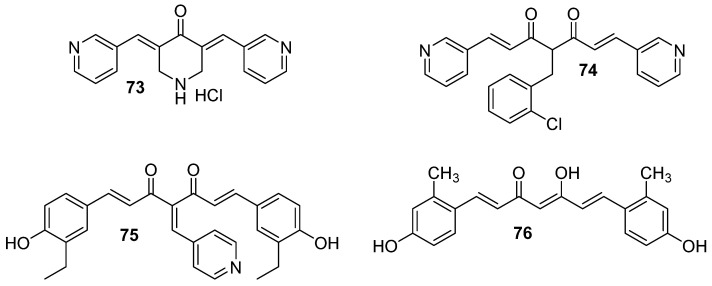
Chemical structures of curcumin derivatives **73**–**76**.

Among modified curcumin derivatives, it is worth mentioning a compound named C817 (**73**, Figure 11), which was tested in vitro on wild-type (WT) and imatinib-resistant mutant Abl kinases, as well as in imatinib-sensitive and resistant CML cells. Compound **73** acted as a potent inhibitor of both WT and mutant Abl kinases, effectively blocking proliferation in vitro. Moreover, it was shown that this derivative could eradicate human leukemia progenitor/stem cells. Thus, this compound might be potentially considered for the treatment of CML patients with Bcr-Abl kinase domain mutations that confer resistance to imatinib [85].

Another curcumin analog that also revealed activity on CML K562 cells and AML HL60 is a compound named C212 (**74**) [86]. It induced apoptosis and cell cycle arrest at the G2/M phase, which inhibited the growing leukemia cells at a higher efficacy than curcumin. Analog **74** (Figure 11) was also responsible for the removal of quiescent leukemia cells, resistant to conventional treatments, which is key to preventing leukemia relapse. Its activity’s mechanism was attributed to inhibiting Hsp90, similarly as it was noted for compound C1205—derivative **75** presented in Figure 11 [79]. Both analogs, **74** and **75**, demonstrated greater activity in Hsp90 inhibition and antitumor effects compared to curcumin. Compound **75** reacted with Hsp90 and degraded protein in both imatinib-sensitive K562 CML cells and imatinib-resistant K562/G01 CML cells. It also suppressed Akt, MEK, ERK, and C-RAF. The result was a significant inhibition of proliferation and induction of apoptosis in K562 and K562/G01 cells.

In another study, curcumin and its derivative, named CD2066 (**76**, Figure 11), exhibited antiviability effects in aggressive T-cell ALL in nanomolar or micromolar concentration, respectively [87]. Both compounds interfered with Notch signaling activity (downregulation), promoted DNA damage, and induced an antiproliferative effect. Among curcumin and seventeen curcumin derivatives, compound **76** was identified as the most active anti-leukemic drug candidate.

Nakamae et al. [88] studied the role of ROS upregulation in tumor suppression. In the study, thirty-nine novel curcumin derivatives were synthesized, and their anti-proliferative and anti-tumorigenic properties were examined. All derivatives exhibited anti-proliferative activity toward human cancer cell lines, including CML-derived K562 leukemic cells in a manner sensitive to an antioxidant, N-acetyl-cysteine. C7-Curcuminoids, **61**, and its demethylated analog **62** were synthesized via aldol condensation of 2,4-pentanedione with 3,5-dimethoxybenzaldehyde (Figure 10). All the C5-curcuminoids were synthesized via the condensation of 1-aryl-1,3-butanediones prepared by the Claisen condensation of the corresponding acetophenone derivatives with ethyl acetate, with aromatic aldehydes. To investigate novel curcumin derivatives’ anti-proliferative activity on human tumor cells, the authors cultured K562 cells in the absence and presence of the representative compounds (50 μM) in vitro. All the compounds showed growth inhibitory activity, but in different degrees, dead cells’ induction varied from one compound to another. Next, the researchers determined and compared the GI_50_ of all curcumin derivatives using K562 cells. Compounds (**61**–**72**) exhibited a GI_50_ lower than that of curcumin in this assay in vitro. The growth inhibitory effect of curcumin derivatives was not restricted to K562 leukemic cells; other types of human cancer cell lines, including U-87 MG glioblastoma, HeLa cervical cancer, MCF-7 breast adenocarcinoma, AN3CA uterine cancer, MIA PaCa-2 and PANC-1 pancreatic cancer, and 293T human embryonic kidney cells, were sensitive to the inhibition of growth by curcumin derivatives.

Suppression of the tumorigenic cell growth of human cancer cells (K562 leukemic cells) in a xenograft mouse model was also studied with curcumin derivatives. Compounds: **62**, **65**–**67**, and **69** significantly reduced the size of tumors, yet the effect was not as pronounced as for curcumin itself. Notably, curcumin and its derivatives did not induce any obvious adverse effects in the normal lineage of cells under the conditions in which curcumin and a group of curcumin derivatives sufficiently inhibit tumor cell growth in vivo, as well as no toxic effects were observed in mice [88].

**Figure 12:**
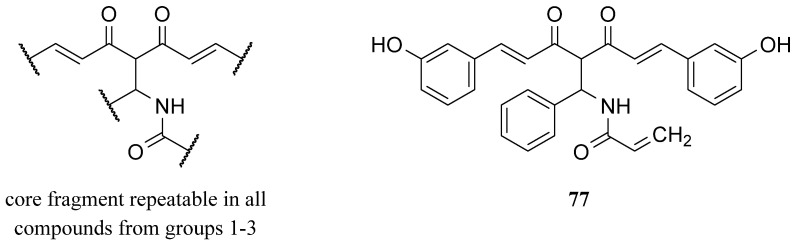
Chemical structures of curcumin derivatives (**groups 1**–**3**, **77**).

The first evidence of an increased incidence of lung cancer was noted among miners and other occupational groups during the 19th century. In the first half of the 20th century, an epidemic rise in lung cancer cases was observed. Today, lung cancer is the most common malignancy among men in many countries and remains the leading cause of cancer-related deaths worldwide. Histologically and biologically, lung cancer is a highly complex neoplasm. While sequential premalignant lesions have been well-defined for centrally arising squamous cell carcinomas, they are less well-documented for other major subtypes, including small-cell lung cancer and adenocarcinoma. The three main morphological types of premalignant lesions identified in the lung are squamous dysplasia, atypical adenomatous hyperplasia, and diffuse idiopathic pulmonary neuroendocrine cell hyperplasia [15,22]. Studies on the pharmacokinetics and bioavailability of curcumin have shown that although the substance is safe and well tolerated even in very high doses, its bioavailability is limited by poor absorption and rapid elimination from the body. According to FAO/WHO recommendations, the maximum daily intake of curcumin is 0–1 mg/kg body weight, which does not cause any adverse health effects. Wahlstrom and Blennow’s pioneering 1978 study on rats showed that curcumin is mainly excreted unchanged in feces after oral and intraperitoneal administration. Some curcumin was found to appear in the bile after intravenous administration, with the main metabolites being the glucuronides tetrahydrocurcumin (THC) and hexahydrocurcumin. These studies have also shown that curcumin accumulates in the intestines and liver but is only found in trace amounts in the brain. Subsequent animal and human studies have confirmed these results, showing low levels of curcumin in the blood after oral administration. In clinical trials in cancer patients given curcumin in high doses, blood levels of the substance were low, while it reached higher levels in intestinal tissues and liver. Despite numerous studies on curcumin’s safety and efficacy, its poor bioavailability limits its use as a therapeutic agent. To overcome these limitations, researchers are testing various methods to increase curcumin’s bioavailability. These include adjuvants that block metabolic pathways, nanocurcumin, liposomes, micelles, and structural modifications such as isomerization. Innovative approaches, such as polymeric nanoparticles, have shown increased efficacy in delivering curcumin to the body. Curcumin analogs improved bioavailability and stronger anti-inflammatory and anti-tumor effects. Despite these promising results, further research is still needed on curcumin’s bioavailability and metabolism, as well as its therapeutic application. Long-term studies on its effectiveness in treating cancer and other conditions are ongoing at various research centers around the world [89] Gyuris et al. performed in vitro cytotoxicity assays with two different lung cancer cell lines (A549 and H1975) to evaluate the new derivatives’ anticancer activities (46 compounds, divided into three series, Figure 12) [90]. The effects of the most potent analogs (most notably **77**) were tested against subcutaneously implanted human lung cancer (A549) in the SCID mouse xenograft model, showing significantly reduced tumor growth.

Curcumin and its derivatives are also being evaluated for their potential use in the treatment of bladder cancer, a common malignancy of the urinary system arising in the tissues of the urinary bladder. Urothelial carcinoma is the most common type of bladder cancer, accounting for more than 90% of cases in industrialized countries. It is particularly common among the elderly, and risk factors include smoking, exposure to chemicals and chronic cystitis. Treatment primarily involves transurethral resection and intravesical infusion of chemotherapy, but may also include laser ablation, Bacillus Calmette-Guerin bladder treatment, radiation therapy, chemotherapy or surgical removal of part or all of the bladder. Natural curcuminoid mixture (curcumin, demethoxycurcumin and bisdemethoxycurcumin) presents activity towards bladder cancer as it inhibits cell proliferation and migration, while promoting apoptosis through the suppression of MMP signaling pathways [91]. Curcumin itself was also found to reduce bladder cancer tumor growth in animal models [92].

**Figure 13:**
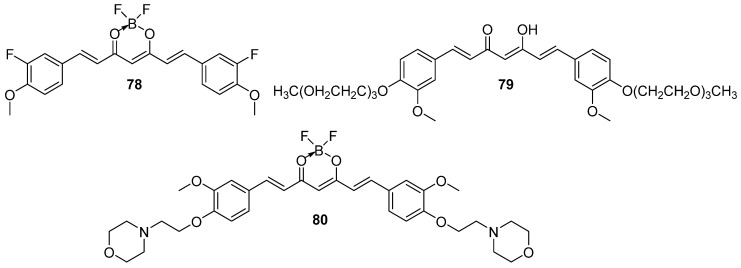
Chemical structure of curcumin derivatives **78–80**.

One potential approach to increasing biological activity is the introduction of fluorine atoms into a molecule. Based on this principle, our group synthesized a series of fourteen curcumin fluoro-analogs [93]. These compounds were tested in vitro against bladder cancer cell lines 5637 and SCaBER. The study showed that the presence of the BF_2_ group at the diketone fragment is crucial for cytotoxic activity, as compounds with an unaltered 3,5-diketone unit showed significantly lower activity against the bladder cancer cell lines. Additionally, curcumin-BF_2_ adducts with a methoxyl group demonstrated higher activity compared to those with hydroxyl or fluorine groups, and compounds with a single fluorine atom were more effective than those with two fluorine atoms. It was also found that the distribution of substituents in the benzene ring significantly affects the anticancer activity of curcumin analogs. Among the synthesized BF_2_ adducts, derivatives with 3-fluoro-4-methoxyphenyl group proved to be the most cytotoxic. Compound **78** (Figure 13) exhibited IC_50_ values of 6.49 μM and 3.31 μM for the 5637 and SCaBER cell lines, respectively, after a 24-hour incubation, demonstrating superior efficacy compared to curcumin.

The introduction of a BF_2_ moiety to the carbonyl groups was also applied by Lazewski et al. [94]. The authors obtained a series of curcumin derivatives by substituting the phenolic groups with poly(ethylene glycol) (PEG) chains and adding a BF_2_ moiety to the carbonyl groups. The compounds were tested for their cytotoxic activity against two bladder cancer cell lines, 5637 and SCaBER. Cell viability was analyzed under normoxic and hypoxic conditions (1% oxygen). The study showed that in a concentration-dependent manner, PEGylated curcumin inhibited the cell cycle in the G2/M phase and induced the expression of proteins involved in cell cycle regulation, cell proliferation and response to hypoxic conditions. Compound **79** (Figure 13) under hypoxia, but not normoxia, increased the expression of stress-related proteins associated with c-Jun N-terminal kinase signaling, angiogenesis, ECM patterning and the p21 signaling pathway.

To improve the pharmacokinetic properties and enhance the biological effects of curcumin, Bakun et al. [24] synthesized and characterized a series of 30 compounds inspired by its structure, which were evaleated towards bladder cancer cell lines 5637 and SCaBER. Compound **80** (Figure 13) was proven to have the best activity, showing IC_50_ values of 1.2 μM and 2.2 μM against 5637 and SCaBER cell lines, respectively, after 24 hours. Analysis of the structure-activity relationship of the most active compounds showed that symmetric curcuminoids exhibited higher anti-tumor activity compared to quasi-curcuminoids. Moreover, modification of curcumin’s β-diketone moiety with the BF_2_ grouping significantly enhanced its cytotoxic activity.

Summarizing the above-discussed publications, one can draw conclusions about how specific structural modifications concerning the hydroxyl and methoxyl groups or ß-diketo moiety influence anticancer activity.

– Hydroxyl and methoxyl groups:
  - Modifications in methylation patterns, such as the addition of a third hydroxyl or methoxyl group, as well as complete methylation or demethylation, did not result in enhanced activity against CML-derived K562 leukemic cells [78].
  - Curcumin-BF_2_ adducts containing a methoxyl group exhibited greater activity against bladder cancer cells (5637 and SCaBER) than those with hydroxyl or fluorine groups [93].
– ß-Diketo moiety:
  - Pyrimidinone derivatives demonstrated improved anticancer activity, and the incorporation of bulky N-substitutions should be considered when designing proteasome inhibitors derived from curcumin [69].
  - Shortening the alkyl chain did not lead to increased activity against CML-derived K562 leukemic cells [78].
  - Complexation of diketone moiety with a BF_2_ group increased cytotoxicity against SCaBER and 5637—bladder cancer lines [24,93,94].

## 3. The Impact of Curcumin Molecule Modifications on the Observed Anticancer Activity

Curcumin, a natural compound found in turmeric, has demonstrated significant anticancer properties, primarily by modulating cell signaling pathways and inducing apoptosis in various cancer types. However, its clinical application is limited due to poor solubility, low stability, and reduced bioavailability. Recent research has focused on structural modifications of the curcumin molecule, such as altering functional groups or introducing substituents, to enhance its pharmacokinetic properties and improve its anticancer potency and selectivity. Curcumin not only reveals an anti-tumor effect but also reverses the effect of multidrug resistance (MDR) in tumor cells. When used in combination therapy, curcumin can act as a factor that sensitizes neoplastic cells to the action of anticancer drugs, which may result in an increase in their effectiveness. The antitumor activity of curcumin measured by the IC_50_ value, depending on the type of tumor and the cell line used in the study, most often ranges from 2–50 µM (colon cancer, breast cancer, ovarian cancer, liver cancer, gastric cancer, lung cancer, human esophageal carcinoma, pancreatic cancer, osteosarcoma cell carcinoma). The effect of reducing the MDR phenomenon is also observed in the case of curcumin derivatives. Changes in the curcumin structure consist of the modification of the side chain on the benzene ring, hydrogenation of the seven-carbon chain, replacement of the β-diketone structure with, e.g., an isoxazole or pyrazole ring, complex compounds, hydrogen substitution of the methylene bridge, replacement of benzene rings with other aromatic heterocyclic rings. Complex modifications include the so-called mixed modifications combining all the previous ones [95].

### 3.1. Modifications of Substituents on Benzene Rings or Hydrogenations of Alkene Chains

**Figure 14:**
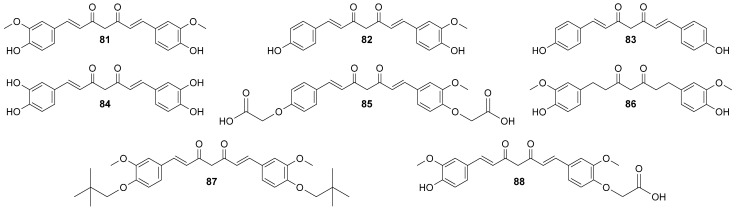
Chemical structures of curcumin derivatives **81**–**88**.

Curcumin ester or demethoxy derivatives were characterized by better stability, and their more favorable antitumor activity was explained, among others, by induction of rapid double-strand breaks of DNA, inhibition of mitosis, and downregulation of P-gp and upregulation of pro-apoptotic signaling (p53/p21 and p16/Rb pathways) [96,97,98,99]. The increase in cytotoxicity of demethoxy curcumin (**82**) observed in studies on colon cancer cell lines (HCT 11 cell) is explained by its greater stability compared to curcumin (**81**) (IC_50_: 3.3, 38.2 µM) [96,100,101]. Both **70**, **82**, and **83** (bisdemetoxycurcumin) demonstrated efficacy against vincristine-resistant (Kb-v1 cell; IC_50_: 23.5, 35.8, 93.0µM, respectively) and wild-type (Kb-3-1 cell; IC_50_: 24.0, 33.3, 85.0 µM, respectively) sensitive cervical cancer cells [96,102,103]. The affinity of curcumin derivatives for aldehyde dehydrogenase-1 (ALDH-1) (**86** > **70** > **81** > **83** > **82**) and GSK-3β (**84** > **81** > **86** > **85** > **82** > **83**) was also observed in breast cancer (Figure 14). In contrast, the introduction of 4 ether groups (**87**, Figure 14) resulted in cell cycle inhibition in the G2/M phase and apoptosis in chronic lymphocytic leukemia cells (K562dox, MDR cell line with high P-gp expression; K562, CML cell line) and simultaneous activation caspase 3 and decreased parp-1 and P-gp levels. For this curcumin derivative, a 10-fold greater anti-tumor and anti-p-gp activity was observed than for curcumin. Thus, such modifications have a positive effect on MDR inhibition [96,104,105]. The substitution of the heterocyclic ring in place of benzene one included the replacement of phenol with a furan ring to increase bioavailability and anti-tumor activity. The results of cytotoxicity studies indicated that such modifications may reverse MDR in a different way, i.e., by lowering the level of the MDR protein Trx in lung cancer cells [96,106,107,108].

Tetrahydrocurcumin (compound **86**, Figure 14) is a carbon chain hydrogenation product and is more hydrophilic and less photosensitizing than curcumin, which facilitates its water solubility, delivery to cancer cells and increases its effectiveness as a free radical scavenger. Therefore, THC may also be a potential MDR reversal agent with the function of modulating the three-drug transporters ABC: ABCB1, ABCG2, and ABCC1 in human cervical cancer. Moreover, THC inhibits caspase-3 activity and levels of protein X associated with B-cell lymphoma 2, induces autophagy in human myeloid leukemia (Ara-C-resistant HL60 cell), influences CSCs suppression and regulation of apoptosis in esophageal squamous cell carcinoma (TE-1 cells resistant to 5-FU), increases the accumulation of Rh123 and calcein in breast and cervical cancer cells (Kb v-1 and MRP1-HEK293 without affecting Kb 3-1 cells) and increases the concentrations of etoposide, mitoxantrone, and vinblastine in cells [96,109,110]. Lai et al. confirmed the chemo-preventive properties of tetrahydrocurcumin in the prophylaxis of colon cancer. Compound **83** showed pro-apoptotic activity through suppression of Wnt-1, expression of the β-catenin protein, GSK-3β phosphorylation, and reduction of the connexin-43 protein level. As a result, inhibition of colon polyp formation was observed by limiting the formation of gap junctions [111,112].

### 3.2. Modifications of Diketone Systems

**Figure 15:**
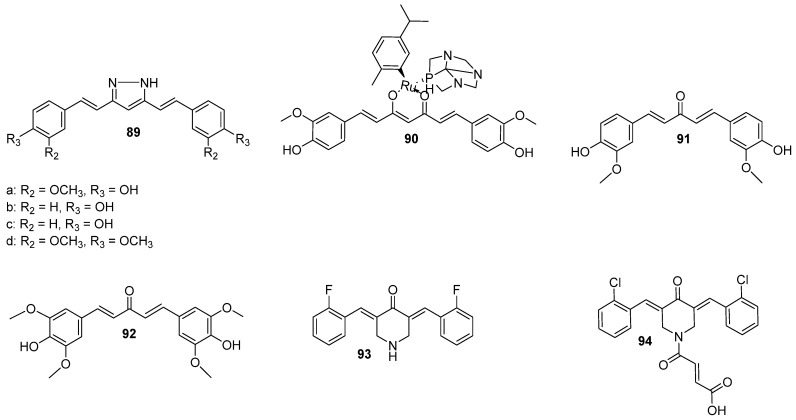
Chemical structures of curcumin derivatives **89**–**94**.

Modifying the diketone system as a form of molecular stabilization leading to an enhancement of the MDR inversion effect has proved to be technically difficult. However, the introduction of a pyrazole ring at this point resulted in the inhibition rate of MCF-7/HER18 cells and MDA-MB 435/HER2 cells being over 40% higher than that of curcumin. These data suggest that this derivative can reverse the MDR of two types of cell lines by reducing HER2 protein expression and blocking the breast cancer cell cycle at the G2/M stage. Other modifications resulted in the observed ability to induce cell apoptosis by reducing the activation of NF-κB and its anti-apoptotic factors (Bcl-2, Bcl-x, survivin, and XIAP in HA22T/VGH and MCF-7/R cells) [96,113,114]. Among the 20 newly obtained curcuminoids and their pyrazole-modified analogs, synthesized by Pham et al., [115] curcumin derivative (**89a**, IC_50_ = 1.53 μM) revealed the highest antitumor activity (liver cancer cell line HepG2). Curcuminoids with a pyrazole ring (**89a**–**89d**, Figure 15) revealed 2–23 times higher antitumor activity compared to their parent structures. The introduction of a fluorine atom as a substituent in the ring significantly weakened or deprived the tested compounds of the desired activity. The authors indicated that hydroxylation of curcumin at position 3 alone increased in activity (IC_50_ = 35.47 µM) compared to curcumin (IC_50_ = 20.70 µM). Curcuminoids acted as Michael acceptors that reacted with GST and GSH in the cell. In contrast, pyrazole analogs were not susceptible to nucleophilic additions with -SH groups in the detoxification mechanism.

The elimination of the ketone group, together with the shortening of the alkyl chain of curcumin, led to the preparation of compounds **91** and **92** (Figure 15), which showed a cytotoxic effect on various human breast cancer cells (MCF-7: ER+, ER−). The obtained IC_50_ values for **91** and **92** were 2.4 and 1.7 µM and could be compared to curcumin (1–7.5 µM) [116,117]. Similar observations were made by Azzi et al., who found EC_50_ values for compound **91** to be 2.9 µM and 6.4 µM in studies on MCF7 and OVCAR-3 cell lines, respectively [118]. Cridge et al. showed that these compounds inhibited Akt, STAT3, and HER2/Neu and activated the process of apoptosis. Their synergy with doxorubicin was also found [116,117].

**Figure 16:**
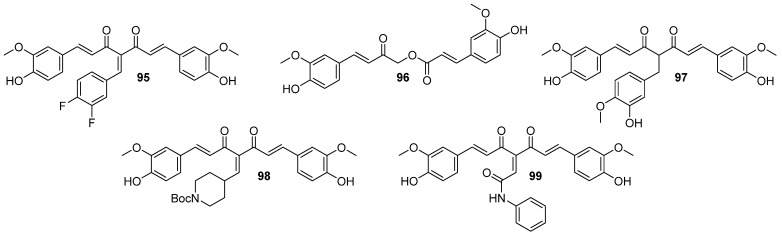
Chemical structures of curcumin derivatives **95**–**99**.

The molecular action of curcumin is related to the removal of reactive oxygen species (ROS) responsible for cell damage. The antioxidant function is given to the curcumin molecule by a diketone residue and two oxidizable phenolic groups and methoxy groups as necessary for the antioxidant action. The study on *D. discoideum* showed the highest and the fastest antioxidant activity of compounds **88** (Figure 14) and **3** (Figure 3). The effect of curcumin lasted for a long time. The remaining compounds revealed significantly reduced or no antioxidant activity. Likewise, the results for curcumin and its derivatives regarding anti-inflammatory activity and antiproliferative disorders did not relate to their ability to modulate ROS. Cocorocchio et al. [117] showed that the action of curcumin derivatives is related to the regulation of cell activity by direct binding of the psrA protein. The authors observed the loss of this protein in the cases of **81**, **82** (Figure 14), and **93** (Figure 15). The gene of *D. discoideum* psrA encodes the ortholog of the regulatory subunit B56 of mammalian protein phosphatase 2A (PP2A). In *D. discoideum*, this protein has been shown to regulate cell chemotaxis and differentiation by negatively modulating the function of glycogen synthase 3 (GSK3) kinase [119]. Compound **91** (Figure 15), which contains two hydroxyl residues, presented better performance than curcumin. The presence of the second hydroxyl group was supposed to increase the binding efficiency of the compound with β-amyloid aggregates. An example of an effective modification of compound **91** is its derivative **94** (Figure 15)—the *N*-maleic acid derivative with strong antiproliferative activity on the H441 lung adenocarcinoma cell line (IC_50_ = 1 µM) [120]. The molecular basis of its anti-cancer activity was related to the presence of the second α, β-unsaturated carbonyl functional group. The ability of compound **89** to deregulate cellular expression of genes and signaling pathways involved in redox processes and glutamate metabolism, leading to increased oxidative stress and cell death, was observed. Compound **95** (Figure 16) also exerted the inducing effect of Nrf2, consistent with the activation of the phase II response involved in the protection of cells from cytotoxicity of oxidative stress. To develop a suitable platform for parenteral delivery, the highly hydrophobic molecule **94** was complexed with cyclodextrins and incorporated into liposomes. In vivo studies in rats showed that the tumor volume was reduced to about half of its original size after 20 days of administration of **95** in liposomes [120].

Derivatives, which constitute curcumin-Ru complexes of the structure shown in Figure 15 (compound **90**), inhibit the effect of P-gp on MDR reversal, which may be of importance in the treatment of ovarian cancer as an alternative to platinum complexes [96,121]. The obtained Ru^II^-arene complexes are air-stable in solution and solid-state and are well soluble in most organic solvents. The antitumor activity of ruthenium(II) aromatic derivatives (p-cymene, benzene, hexamethylbenzene) containing modified curcumin ligands was assessed using five tumor cell lines. The best activities [IC_50_ (µM)] were observed for breast cancer cell lines (MCF7, IC_50_ 9.7 µM), ovarian cancer (A2780, IC_50_ 9.4 µM), glioblastoma multiforme (U-87, IC_50_ 9.4 µM), and lung cancer (A549, IC_50_ 13.7 µM) and colon cancer (HCT116, IC_50_ 15.5 µM). The anti-tumor activity of this group of compounds was manifested by their pro-apoptotic activity. This effect was approximately twice as strong as that of the corresponding curcumin complex in breast and ovarian cancer cells. The replacement of the hydroxyl groups of curcumin with methoxyl groups in the complexes resulted in the observed increase in antitumor activity. Their increased cytotoxicity to cancer cells correlates with the increased lipophilicity of curcuminoid. These compounds do not contain hydroxyl groups, which are responsible for the antioxidant properties of ligands. Therefore, the antioxidant properties were not observed in the described Ru^II^ complexes. Carruso et al. [122] showed that with only one exception (ligand CurcII), the chemical substitution on the backbone of curcumin seems to produce a biologically more effective molecule, which presents lower IC_50_ than their Ru(II) complexes.

### 3.3. Modifications of a Methylene Group

Among the curcumin derivatives obtained by modification of active methylene, two deserve attention (Figure 16). Compound **95** (CDF) inactivated mir-21, which led to the reactivation of PTEN-tumor suppressor proteins mir-200b and mir-200c and inactivation of the phosphorylated material. Moreover, in colon cancer, CDF inhibited the MDR of 5-FU and oxaliplatin-resistant colon cancer cells by triggering the mir-21-pten-akt pathway [96,105,123]. In contrast, in the cells of the chemo-resistant human colon cancer cell line (HCT116CR), CDF increased the expression of mir-34a and mir-34c, which may also inhibit the proliferation of human prostate cancer cells. In contrast, the replacement of the methylene group with a methyleneoxy group (compound **96**) resulted in an increase in the activity of the 38 kDa protein kinase (p38), a decrease in the activity of c-jun-terminal kinase (Jnk) and signal-regulated extracellular kinase (Erk), and inhibition of the effect of P-gp on MDR without affecting the expression level of this protein in gastric, lung and liver cancer [96,124]. The introduction of a 3-methoxy-4-hydroxybenzyl (compound **97**) substituent into the methylene chain led to an antiproliferative active compound against KM12 and SW480 mouse colon cancer cells. This effect was dose- and time-dependent and was likely due to inhibition of NF-κB activity by inhibiting IκBα phosphorylation. The observed cell growth suppression was probably five to seven times greater than the effect of curcumin [111,125].

In turn, a strong anti-tumor effect was observed when testing compound **98** (Figure 16) equipped with an *N*-substituted piperidine ring. The compound showed antiproliferative activity against breast cancer cells (MCF-7; IC_50_ 1.5 µM) [116,126].

In the context of its multidirectional action, compound **99** constitutes an example of a curcumin analog originally designed as a metalloproteinase inhibitor with anti-inflammatory properties. However, this compound, in combination with SBT-1214 (a new-generation taxoid), contributed significantly to the deaths of highly metastatic CSC cells of the prostate and colon. This effect was much stronger than when the compounds were used separately [120].

### 3.4. Mixed Modifications and Hybrids

**Figure 17:**
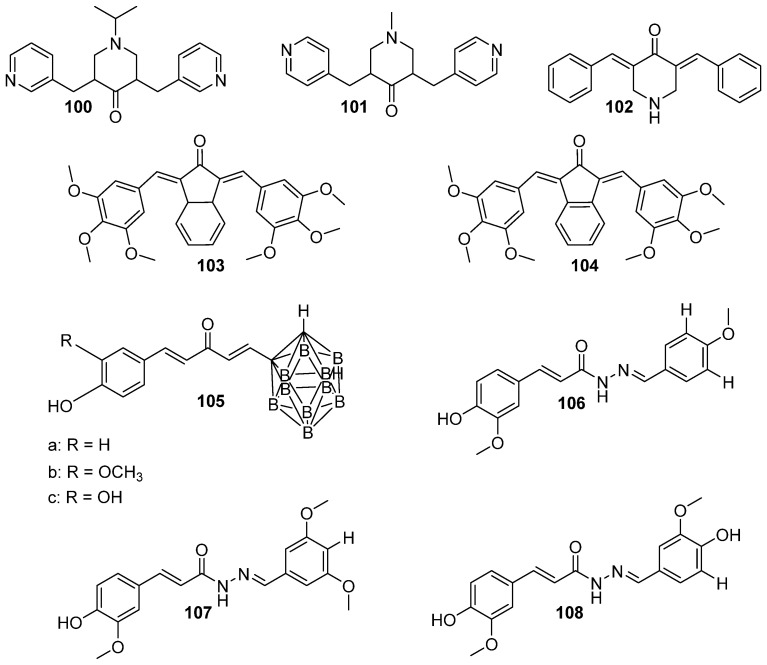
Chemical structures of curcumin derivatives **100**–**108**.

The introduction of the 4-ketopiperidine ring instead of the diketone moiety (Figure 17, compounds **100** and **101**) resulted in the preparation of anti-tumor active derivatives. Their cytotoxicity was confirmed for lung adenocarcinoma cells (ALK^+^ H1322) in the sub-micromolar range. These derivatives, compared to crizotinib, presented little or no direct inhibitory effect on ALK. Thus, since the curcumin derivatives **100** and **101** and crizotinib acted independently, combination therapy might be an effective lung cancer treatment strategy. The compound **101** (cell line H3122, IC_50_ = 0.7 µM) proved to be particularly promising. In contrast, **102** (cell line H3122, IC_50_ = 1.1 μM) also proved to be effective and non-toxic in breast cancer xenograft models, which justifies the interest in this type of modification of the curcumin molecule [127]. Curcumin analog—compound **102** (Figure 17) showed increased ROS production and decreased oxygen consumption in HCT-116 colon cancer cells. Derivatives of compound **103** exhibited antiproliferative activity by interfering with mitochondrial function also against HCT116 and HT29 cell lines, achieving IC_50_ values ranging from sub-micromolar to nano-molar. It was shown that it was the amide carbonyl groups that significantly contributed to the cytotoxic activity of these derivatives [111,128,129]. The curcumin analog **103**, containing the inden-2-one ring, was active against prostate cancer BxPC-3 cells, pancreatic cancer, HT-29 colon cancer cells, H1299 lung cancer cells, and non-cancerous human prostate epithelial cells (RWPE-1). Its cytotoxic and antiproliferative activity against all cell lines was 20 times stronger than that of curcumin [111,130].

The inden-2-one derivative (compound **101**, Figure 17) was effective in an anti-tumor study using prostate cancer cells (PC-3; IC_50_ 0.64 µM). In the same study, curcumin reached an IC_50_ of 19.98 µM. The IC_50_ value of compound **102** in RWPE-1 cells was higher than in PC-3 cells, indicating that compound **101** is more toxic to cancer cells than to non-cancer cells [116,130].

Initial in vitro studies of novel boron derivatives (**105a**–**c**, Figure 17) that resulted from the replacement of the aromatic ring with an ortho-carborane cage showed significant cytotoxic activity, with EC_50_ values ranging from 1.8 to 5.5 µM. The studies were performed with the use of MCF7 and OVCAR-3 (human ovarian adenocarcinoma) cell lines. These compounds additionally inhibited the formation of β-amyloid aggregates seen in Alzheimer’s disease [118].

Another approach in the synthesis of curcumin derivatives is the synthesis of curcumin-resveratrol hybrids (**106**–**108**) (Figure 17). In studies on three tumor cell lines MCF-7 (breast), A549 (lung) and HepG2 (liver), their cytotoxic effect were confirmed by observing lower IC_50_ values on MCF-7 cells (IC_50_: **106** 40.49 µM; **107** 19.09 µM; **108** 42.99 µM) compared to the effects of curcumin (IC_50_ 68.25 µM), resveratrol (IC_50_ 128.85 µM) or combined administration of these compounds (IC_50_ 61.71 µM). In the treated cells, the authors observed a decrease in the G0/G1 and S populations while an increase in the G2/M population. A significant increase in CDKN1A mRNA was also noted in the samples treated with **104** compared to the samples treated with the combined use of resveratrol and curcumin at the same concentration. Thus, the effect of the hybrid in promoting p21 regulation in MCF-7 cells is more potent compared to the combined use of resveratrol and curcumin. The p21 protein belongs to the Cip/Kip family of proteins that promote cell cycle arrest by binding to cyclin-dependent kinases (CDKs). The significant reduction in mRNA abundance for the three mitotic kinases (aurora A, aurora B, and PLK1) in the **107**-treated cultures compared to the control group was less than when compared to the combined use of curcumin and resveratrol but sufficient to inhibit mitosis [131].

## 4. Conclusions and Perspectives

**Figure 18:**
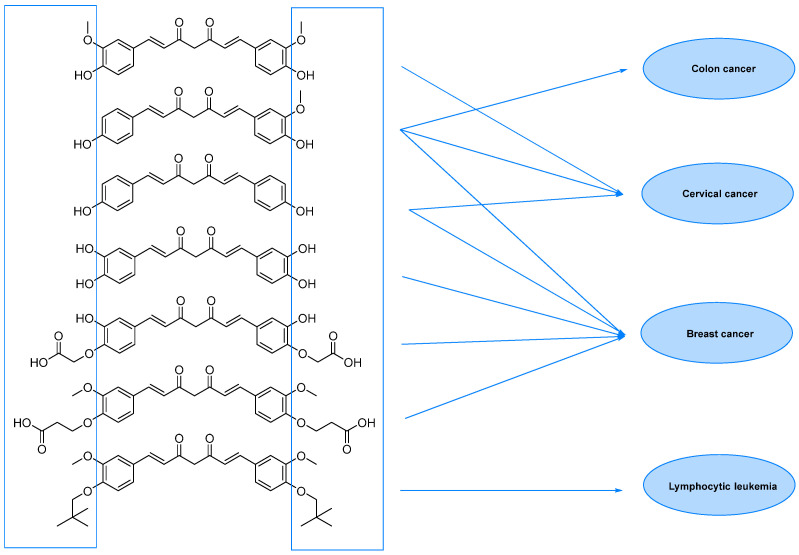
Structural modifications of curcuminoids active against cancer.

The curcuminoid structure has become the starting point for the development of compounds with a wide range of activities: antitumor, anti-inflammatory, compounds effective in the treatment of neurodegenerative diseases, etc. Literature data confirm the growing interest in this polyphenol and indicate its numerous benefits. Thus, sensible structural modifications, combined with innovative technological strategies, will overcome some of curcumin’s limitations, such as poor stability and low solubility in physiological conditions that preclude its clinical application (Figure 18). Curcumin reveals antiproliferative activity in many cancers, inhibits transcription factors, modulating the activity of growth factor receptors and cell adhesion molecules involved in angiogenesis, tumor growth, and metastasis. There is also the possibility of its influence on the inhibition of telomerase. Meanwhile, curcuminoids show a bifunctional effect by blocking the anti-apoptotic signaling of NF-κB but also by blocking the anti-oncogenic effect of STAT-1 and the production of interferon-γ. In contrast, Ga-curcuminoid complexes showed potential for use as radiotracers in the detection of lung cancer [120,132]. Curcumin derivatives and analogs also constitute a widely studied group of compounds with anti-inflammatory properties. The most important molecular target of their action is NF-κB responsible for the regulation of the immune and inflammatory responses. Some derivatives revealed both anti-proliferative and anti-inflammatory effects. Chainoglou et al. [131] considered the basic features linking the activity of this group of compounds with the molecular target or signaling pathway: the presence of heterocyclic aromatic rings (thienyl, pyrazolyl, pyrimidinyl), hydroxyl and methoxy groups, removal of substituents, the introduction of lipophilic substituents, bromines, diaryl pentanoid rings or carbon linkers to increase antiproliferative activity. Hybridization also appears to have an impact on these properties [133].

**Figure 19:**
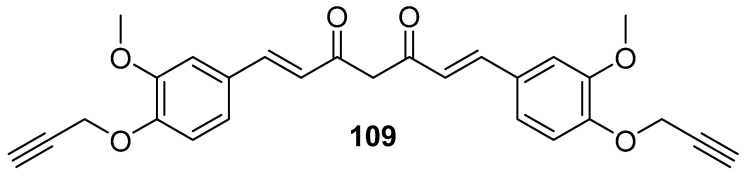
Chemical structure of curcumin analog **109**.

Curcumin not only has an anti-tumor effect but also reverses the effect of MDR in tumor cells. In combination therapy, curcumin, in combination with chemotherapeutic agents, can act as a factor that sensitizes neoplastic cells to the action of anticancer drugs, which may result in an increase in their effectiveness. The antitumor activity of curcumin measured by the IC_50_ value, depending on the type of tumor and the cell line used in the study, most often ranges from 2–50 µM (colon cancer, breast cancer, ovarian cancer, liver cancer, gastric cancer, lung cancer, human esophageal carcinoma, pancreatic cancer, osteosarcoma) [95]. The observed effect of reversing the MDR phenomenon in the presence of curcuminoids makes it possible to search for new curcumin derivatives and to create combinations with other chemotherapeutic agents. Changes in the curcumin structure consist of the modification of the side chain on the benzene ring, hydrogenation of the seven-carbon chain, replacement of the β-diketone structure with the heterocyclic ring, obtaining complexes, hydrogen substitution of methylene bridge hydrogen, replacement of benzene rings with other aromatic heterocyclic rings. More advanced modifications include the so-called mixed modifications combining all the previous ones [95]. Knowing that curcumin itself exhibits anticancer activity and may additionally help overcome the multidrug resistance of cancer cells by inhibition of the P-gp, Lopes-Rodrigues et al. [9] synthesized a series of curcumin derivatives. First, they were first assessed for their anticancer potential towards K562 (chronic myeloid leukemia) and NCI-H460 cells (non-small cell lung cancer), and their multidrug-resistant analogs overexpressing P-gp, K562Dox, and RH460, respectively. The best activity in this manner, as compared to curcumin, was expressed by **109** (Figure 19) and **91** (Figure 15). Interestingly, **109** was more potent in the drug-resistant cells.

**Figure 20:**
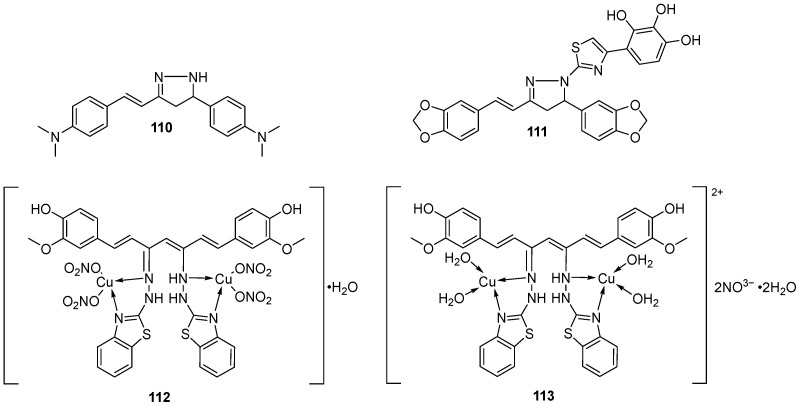
Chemical structures of compounds **110**–**113**.

It is also important to be aware of the interesting and important preventive, especially antioxidant potential of these compounds. This is because ROS are formed by oxidation reactions in organisms. Biochemical imbalances caused by ROS can damage many biological macromolecules, including DNA, RNA, lipids, and proteins, leading to degenerative diseases such as multiple sclerosis, cancer, and Alzheimer’s disease. Curcuminoids exhibit strong antioxidant activity, surpassing α-tocopherol, a well-known natural antioxidant. This potency is attributed to its ability to neutralize harmful ROS, such as superoxide anions and hydroxyl radicals. Curcumin can protect cells from DNA damage caused by lipid peroxides and singlet oxygen and may also impact neurodegenerative diseases and atherosclerosis. Its neuroprotective effects stem from its antioxidant capabilities, as neurodegeneration often results from oxidative damage by ROS and reactive nitrogen species (RNS). Elevated protein oxidation and oxidative DNA damage are common in neurodegenerative conditions. Despite its benefits, curcumin’s poor bioavailability hampers its medicinal use, leading scientists to develop analogs to enhance its antioxidant properties [134,135,136,137]. The antioxidant activities of many curcumin derivatives, like **109** (Figure 19) and **110**–**113** (Figure 20), have also been intensively studied [136,137,138].

The difficulty in determining the optimal modifications of curcumin lies in its greatest benefit— curcumin does not exert its activity through a single cellular pathway, but it has the ability to simultaneously activate or deactivate several targets. Thus, the observed effects for a given derivative may be a result of tuning the structure to be more specific to a particular target rather than slightly potentiating all pathways. Additionally, curcumin has three motifs that may be responsible for its activity—the β-diketone, substituted aromatic rings, and Michael acceptor fragment, with the latter capable of blurring the picture due to non-specific action. Thus, caution should be taken when interpreting the results of the biological activity of curcumin derivatives.

To sum up, by functionalizing the parent curcumin molecule, researchers have obtained more stable and bioavailable compounds with enhanced therapeutic potential, making curcumin derivatives promising candidates for medical applications, including cancer. Apart from the anticancer activity of new curcuminoid derivatives, it is worth paying attention to their high protective potential against free radicals and the possibility of use in combined therapies, especially in cases of multidrug resistance.

## Data Availability

Not applicable.
